# Plasma-treated water applied as a foliar spray promotes root growth in barley

**DOI:** 10.1186/s12870-025-07394-w

**Published:** 2025-09-22

**Authors:** Andrea Krüger, Stefan Schlömer, Stefan Simm, Jessica Bold, Christine Stöhr

**Affiliations:** 1 Institute of Botany and Landscape Ecology, University of Greifswald, Greifswald, 17489 Germany; 2 Institute for Bioinformatics, University Medicine Greifswald, Greifswald, 17475 Germany; 3 Institute for Bioanalysis, Department of Applied Sciences, Coburg University of Applied Sciences and Arts, Coburg, 96450 Germany

**Keywords:** Cell elongation, *Hordeum vulgare*, Plasma-treated water, RNA-seq, Root architecture

## Abstract

**Background:**

Plasma-treated water (PTW) contains reactive oxygen and nitrogen species and can therefore induce mild oxidative stress in plants. In a previous study, PTW treatment of leaves resulted in both short- and long-term systemic signalling effects. In this study, we analysed the adaptive response of the barley leaf and root transcriptome triggered by PTW treatment under short- and long-term conditions to further understand the regulatory mechanisms and the mode of action of PTW.

**Results:**

The application of PTW to the leaves resulted in stronger long-term transcriptional changes in the roots. PTW treatment significantly increased root biomass, while it had no effect on shoot growth. In roots, the altered gene expression indicated that the signalling of auxin, ethylene, brassinosteroids and gibberellin was stimulated, while cytokinin signalling was impeded. Many genes encoding cell wall modifying enzymes, structural proteins and receptor kinases were upregulated in the roots in long-term response to PTW. In addition, foliar treatment with PTW led to changes in root architecture: root length, surface area, diameter and number of forks increased in the long term, as did the content of soluble phenolic compounds.

**Conclusions:**

All these changes indicate that PTW treatment affected root growth possibly by promoting cell elongation. Thus, PTW could be a suitable stimulant to improve the root growth of plants.

**Supplementary Information:**

The online version contains supplementary material available at 10.1186/s12870-025-07394-w.

## Background

Plasma-treated water (PTW) is produced by a low-temperature plasma source operating under atmospheric air conditions in combination with water. This PTW is a viable option for the controlled generation of a variety of reactive oxygen and nitrogen species (RONS) [1] and has been shown to be a potential priming agent [2]. As membrane lipids are considered the primary target of RONS [3, 4], lipid peroxidation can produce malondialdehyde (MDA) and signalling lipids (e.g. oxylipins). These signalling lipids can activate defence responses at the transcriptional level and act as stress mitigators [5]. In addition, MDA can be a gene activator in stress acclimatisation as long as its concentration is properly balanced [6]. The formation of MDA is a symptom of environmentally stressed plants. Another symptom and defence mechanism is the increased synthesis and structural modification of lignin to prevent membrane damage due to lipid peroxidation [7]. To this end, the phenylpropanoid metabolism is generally stimulated under stress and produces phenolic compounds with antioxidant activity based on the number and localization of functional groups [8], enabling them to quench RONS. The key enzyme phenylalanine ammonia lyase (PAL) is considered an indicator of stress conditions and thus, a biochemical marker indicating the activation of plant defences. Irrigation of tomato plants with PTW for up to 5 weeks enhanced the expression of PAL [9].

As these effects of RONS have already been described, it is also shown that many of the RONS generated in PTW have only a very short half-life, while hydrogen peroxide (H_2_O_2_), nitrite (NO_2_^−^), and nitrate (NO_3_^−^) are frequently detected in conjunction with a drop in pH [10]. This could mean that RONS can act as a priming stimulus leading to increased stress tolerance by inducing a mechanism to remember past stress events and to respond faster or more strongly to recurrent stress in plants [11]. Epigenetic processes determine the durability of this memory. Long-term stress memory is particularly useful for younger plants, as it can help them to allocate their resources between growth and defence [12]. The RONS generated by cold plasma may be involved in the activation of biochemical and molecular processes that promote plant growth and sustainability [13]. PTW induced a rapid and sustained increase in cytosolic Ca^2+^ in *Arabidopsis thaliana*, while the separate administration of nitrate, nitrite, and hydrogen peroxide at the same doses did not induce any detectable Ca^2+^ changes [14]. Thus, the complex mixture of different RONS in PTW is likely the cause of the long-lasting Ca^2+^ elevation and could be a crucial factor for the induction of a primed state.

PTW treatments have been shown to enhance root development and improve nutrient availability and uptake (reviewed by Konchekov et al. [15]). Tobacco plants grown in PTW exhibited an increase in both the length of the primary root and root hairs and in cotyledon size due to cell elongation [16]. Maize and pea plants showed increased root growth after seed treatment with PTW and additional positive growth effects when irrigated with PTW [17]. Wheat grains watered in PTW with a low dose of reactive species displayed significantly stimulated root and shoot length growth [18]. Root and shoot length of barley seedlings increased significantly compared to the control after growing in PTW for 18 days [19]. While most studies used seed treatment or irrigation with PTW, one possible method of administering PTW is foliar application to promote uptake of water and solutes through the cuticle or stomata of plant leaves [20]. Penetration of the cuticle could occur through polar pores: at high humidity, water molecules dock to hydrophilic polysaccharides. This leads to swelling of the cuticle and to a continuous connecting water film between the leaf surface and the epidermis, creating pores large enough to allow NO_3_^-^ to pass through. On the other hand, hydrophilic particles can enter by diffusion along the walls of the stomatal pores. Both diffusion ways contribute equally to the penetration of dissolved substances. Wettability is a prerequisite for penetration into the foliage [21]. Plasma activation of water was shown to increase the wettability of a glass surface by lowering the contact angle between the water and the surface increasing the contact area [22]. Furthermore, a possible acidification of the leaf surface can increase the permeability of the cuticle for anions [23].

Previously, we investigated the short- and long-term effects of PTW on the ascorbate-glutathione cycle in barley leaves and roots in the absence of stress and under waterlogging conditions [2]. PTW treatment of leaves affected all components of the ascorbate-glutathione cycle in barley leaves and roots. This systemic signalling effect occurred both in the short- and long-term and was stronger under stress conditions. To further understand the regulatory mechanisms and the mode of action of PTW in this phenotypic behaviour, we used RNA-seq to analyse screenshots of the transcriptome to obtain a more holistic view. Therefore, we investigated changes in terms of short- and long-term differences as well as directly administered organs (leaves) and indirectly affected organs (roots) in barley. In barley, the primary root system composed of embryonically formed primary and seminal roots is small and only important at early developmental stages [24]. Post-embryonically, a fibrous root system develops from shoot-born roots, the so-called adventitious roots or crown roots. All root types have the ability to form lateral roots. Every aspect of root development is linked to the action of auxin. NO and H_2_O_2_ can be downstream signalling molecules in the auxin signalling cascade in the induction of adventitious roots, and NO may be an upstream signal for H_2_O_2_ production [25]. These small molecules promote adventitious root formation by regulating antioxidants, water balance and photosynthetic systems, as well as by influencing auxin distribution. Both NO and H_2_O_2_ are components of the PTW used in this study [26]. Here, a small amount of PTW was applied as a foliar spray to serve as a signal and to avoid a fertilization effect. We intended to validate previous results in this system in order to establish PTW as a robust technology for plant stress priming.

## Materials and methods

### Production of plasma-treated water

The alternating current driven cold atmospheric plasma system used in this study consisted of a pin-to-liquid discharge configuration with four metal electrodes located approximately 3 mm from the water surface [27]. Deionized water was mixed with 7.5% (v/v) tap water prior to plasma treatment, as a sufficient ion concentration (≥ 80 µS cm^−1^) was required to ignite the plasma between the electrodes and the water surface. A water mixture of 900 ml was treated for 20 min and applied to the plants 5–10 min after treatment. This plasma treatment resulted in the accumulation of hydrogen peroxide, nitrite and nitrate ions in µmolar concentrations, and significant amounts of NO were detected released from agitated PTW into the gas phase [26]. The pH value of the PTW was 3.8.

### Plant material and cultivation

The seeds of *Hordeum vulgare* cv. KWS Kosmos were pre-germinated in Petri dishes on filter paper soaked in 0.5 mM calcium sulphate for 2 days in the dark. Each seedling was placed in a pot (⌀ 12 cm) with a homogeneous mixture of coarse-grained and fine-grained quartz sand in a ratio of 2:1 (v/v). The plants were grown in the greenhouse in a light/dark cycle of 14/10 hours at an air temperature of 22/18°C. Depending on weather conditions, sunlight was supplemented with light from high-pressure sodium lamps for the plants grown for RNA-seq and metabolite measurements. For root system architecture analysis, plants were grown under broadband LED lamps (VYPR, Fluence). Pots were rotated twice a week to ensure uniform growing conditions. All plants were watered daily with a defined nutrient solution containing 5 mM nitrate [26]. All experiments were performed in accordance with relevant regulations and guidelines.

### PTW application on barley plants

One group of plants was sprayed with PTW on three consecutive days at the two-leaf stage (BBCH 12 according to Meier [28]). Another group of plants was sprayed with a mixture of deionized water with 7.5% (v/v) tap water (as the water used for PTW generation) instead of PTW as a control on the same three consecutive days. Each treatment consisted of two sprays of water on the leaves from one side and again turned 180°. For the four sprays 1.7 ml of water was used.

### Plant harvest for RNA isolation

Plants were harvested in the morning one day after PTW treatment (short-term, BBCH 12, that was 22 days after sowing) and four weeks after PTW treatment (long-term, BBCH 28, that was 49 days after sowing). The first fully developed leaves and whole roots were pooled from 5 plants each to obtain three biological replicates per group. Samples were shock-frozen in liquid nitrogen and homogenized using a sterile, ice-cooled mortar and pestle. Total RNA was extracted from 100 mg of frozen leaf or 250 mg of frozen root powder using the NucleoSpin RNA Plant and Fungi Kit (Macherey-Nagel, Germany) according to the manufacturer’s instructions. RNA concentration was quantified photometrically using a NanoDrop and RNA integrity was tested on a 1% agarose gel (Supplementary Fig. S1 & S2).

### Plant harvest for metabolite assays

Barley plants were harvested in the morning at BBCH growth stages 12, 22, 26 and 28 [28]. The first and second fully developed leaves and the roots were harvested, ground in liquid nitrogen and stored at − 80 °C. The frozen tissue powder was treated with specific extraction reagents for each assay.

### Biomass and root system architecture

The fresh weight was determined separately for shoot and root after bisecting the plant at BBCH growth stages 22, 26 and 28. Roots were first rinsed in deionized water and attached sand was carefully removed with a soft brush. Then, the roots were cautiously dried prior to weight measurements. For morphological analysis, the root system of a single plant was spread out in a petri dish (⌀ 14 cm) filled with water to avoid excessive overlapping of the roots. In the analysis, nodal and seminal roots were not differentiated. Images were captured with EpsonScan Scanner Perfection with contrast set to −25% and brightness set to −25%. Background noise and grains of sand were removed using GIMP version number 2.10.22. Prior to WinRhizo analysis, the images were colour inverted and rendered with − 50% highlight shaders to obtain black root images on a clear white background. The determination of root hierarchy and architecture was performed with WinRhizo version 2016a Pro. The precision scale was set to standard and crossing detection to normal.

### RNA sequencing and bioinformatic analysis

Total RNA was shipped to Novogene (Cambridge, UK) to generate an mRNA library after poly-A enrichment. High-throughput sequencing was then performed on Illumina NovaSeq 6000 instruments with three replicates per treatment, organ and time point. The sequencing depth was between ~ 39 mio. and 56 mio. read-pairs (paired-end sequencing; Supplementary Table S1). The raw data are available in the GEO under the accession GSE294408 and the Project PRJNA1249405. Mapping of reads to the barley genome (Hordeum Vulgare MorexV3, Phytozome) was performed using NextGenMap [29]. Parameter settings included –very-sensitive and –no-unal beside the standard parameter. In general, ~ 50% of all reads could be mapped to the genome and were used for gene counting using featureCounts (version 2.0.4) [30] via the R package. Parameter settings were adjusted to GTF featureType “gene” and attrType “gene_id” for unstranded reads. The gtf-file was downloaded from the Phytozome (Hordeum Vulgare Morex V3) and used for the counting. About 80% of all mapped reads were assigned to barley genes. About 20,000 genes were found per leaf sample and 23,000 genes per root sample (out of more than 39000 genes in the barley genome according to Hansson et al. [31]).

Differentially expressed genes (DEGs) were determined using DESeq2 [32] with standard parameters between control and PTW treatment independently for organs and time points. The DEGs were divided into PTW upregulated (positive log2FC) and control upregulated (negative log2FC). The use of the R package enhancedVolcano led to the generation of Volcano plots. The mean expressions calculated from the three biological replicates per treatment, organ and time point were used for Principal Component Analysis (PCA). For this analysis R with RStudio was used together with the libraries prcomp and factoextra. The contributions and values of individuals (genes) and variables (samples) to single principal components as well as the variance explanation of principal components were calculated. Visualization of the PCA plots was performed with ggplot (R library) and Sigma Plot (Systat Software GmbH, Erkrath, Germany). When adjusted p-values were set to < 0.05, 12 DEGs were identified in the leaves and 6 DEGs in the roots one day after PTW treatment, while four weeks later, 232 DEGs in leaves and 3460 DEGs in roots were detected. For further DEG analysis the│Log2FC│was set to ≥ 1 (corresponding to a minimum fold change of 2). In this way, the DEGs should be adjusted for most genes that might show mismatched results (as 93% of these genes have a fold change of less than 2 according to Coenye [33]). Non-matching in this context means that DEGs would show different patterns of differential expression when using different methods (e.g., qPCR). A Gene Set Overrepresentation Analysis (GSOA) of the selected DEGs was performed with G: Profiler (https://biit.cs.ut.ee/gprofiler/gost) [34] of the organism *Hordeum vulgare* subsp. *vulgare* (MorexV3_pseudomolecules_assembly) using the False Discovery Rate to select significant gene ontologies (GO) with all expressed genes in both samples of the comparison as a background list. These lists were uploaded to R version 4.2.2 and selected by GO source (bp, cc, mf) with a cutoff of *p* < 0.05. The gene ratio was calculated with intersection size/term size. The data frame was converted to an enrichResult object using the R package multienrichjam (version 0.0.64.900) [35] and dotplots were generated using the dotplot function of the package clusterProfiler [36, 37].

### Measurement of thiobarbituric acid-reactive substances

Lipid peroxidation was determined and calculated in terms of thiobarbituric acid-reactive substances (TBARS) corresponding to MDA equivalents. To measure TBARS, the method described in Savchenko et al. [38] was applied with slight modifications. 0.1 g of frozen leaf or root powder was macerated in 1 ml of 0.5% (w/v) trichloroacetic acid. The samples were incubated on ice for 20 min, and then centrifuged at 18,000 g at 4 °C for 15 min. Subsequently, 0.2 ml of the supernatant was mixed with 0.4 ml of 20% trichloroacetic acid containing 0.65% thiobarbituric acid, and the samples were incubated at 95 °C for 30 min, rapidly cooled on ice, and centrifuged at 15,000 g at 4 °C for 5 min. The absorbance spectrum of the supernatant was measured at 532 nm on a plate reader and corrected for non-specific absorbance by subtracting the value of absorbance at 600 nm and 440 nm [39, 40]. The concentration of TBARS was expressed in nmol g^–1^ FW using the extinction coefficient for MDA of 155 mM^−1^ cm^−1^.

### Measurement of the content of soluble phenolic compounds

The analysis of phenolic compounds was performed using reverse-phase high-performance liquid chromatography (HPLC) with detection at 230 nm. Phenolic compounds were extracted from 0.1 g of frozen leaf or root powder in 1 ml of pure methanol. Sonication was performed at 42 °C and 100% amplitude with activated degas function for 15 min. Partially extracted leaf samples were subjected to two further extraction cycles, averaging about 3 ml for leaves and 1 ml for roots in total. The samples were sedimented by centrifugation at 18,000 g and 4 °C for 5 min. From the pooled extracts, 1 ml of supernatant was evaporated in a vacuum concentrator at room temperature in the dark for 90 min. The residues were mixed with 1 ml of solvent A (0.3% phosphoric acid in ultrapure water), then filtered through 0.22 μm PES filters. The prepared samples were stored on ice in the dark until injection into a Rheodyne sample injector model 7161. The HPLC system setup consisted of a Merk Hitachi L-6200 A Intelligent Pump, coupled with a Merk Hitachi L-4250 UV-Vis detector. A Phenomenex LC Column (Gemini 5 μm C18 110 Å), Size 150 × 4.6 mm was used with a Phenomenex SecurityGuard Cartridge Kit, Analytical Standard KJ0-4282 for analysis. The column oven temperature was maintained at 30 °C at a flow rate of 0.8 ml/min. Running buffers consisted of solvent A and solvent B (80% acetonitrile and 0.3% phosphoric acid in ultrapure water) and were mixed according to a gradient protocol [41]. Data acquisition and analysis was performed using Clarity chromatography software from DataApex, version 8.4.0.47. Compounds were identified and quantified by comparison with authentic pure HPLC-grade standards.

### Determination of soluble protein content

Soluble proteins from 0.25 g of frozen leaf or root powder were extracted in 1 ml of 100 mM MES/KOH buffer (pH 6.0) containing 40 mM KCl, 2 mM CaCl_2_, 2% (w/v) PVPP, and 1 mM ascorbic acid, by incubation on ice for 10 min (occasionally shaking) and centrifugation at 24,000 g at 4 °C for 10 min. The protein content of the supernatant was determined using bovine serum albumin as a reference [42].

### Statistical analysis

Biological replicates are seeds of *Hordeum vulgare* cv. KWS Kosmos. They were pre-germinated at the same time and then separated in distinct pots and treated equally except for the control versus PTW treatment. Metabolite and root system data are presented as mean ± standard deviation (SD). Data were statistically analysed using student’s t test in Excel. For the DEG and GSOA analyses we used Benjamini-Hochberg for adjusting the p-values based on the provided implemented tests by DESeq2 and clusterProfiler.

## Results & discussion

### Mild stress by PTW treatment of leaves led to short-term lipid peroxidation in leaves and roots

The RONS contained in PTW are known to cause mild oxidative damage to macromolecules such as phospholipids [3]. The degree of lipid peroxidation was measured as an indicator of the effect of PTW treatment. Lipid peroxidation in terms of thiobarbituric acid-reactive substances was significantly increased in both leaves and roots one day after PTW treatment (Supplementary Table S2, Supplementary Fig. S3). This indicates both a direct response in the leaves and a systemic signalling effect. As expected, no significant changes in lipid peroxidation were observed four weeks after PTW treatment. Lipids as signalling molecules in the form of oxylipins have been reported to upregulate glutathione S-transferase (GST) and influence ascorbate and glutathione levels [43]. One GST gene *(GSTU20)* was upregulated in roots as a short-term response to PTW treatment and another as a long-term response (Supplementary Table S1). The stimulation of the antioxidant system was demonstrated in a previous study: PTW increased the content of ascorbate and glutathione in barley leaves and roots [2]. In leaves, the lipoxygenase pathway was stimulated in the long term (Fig. 2).

### Gene expression was more differentially regulated in both organs in the long term after PTW treatment

To determine which processes are regulated by PTW in the short and long term we analysed the general transcriptomic adjustment in barley leaves and roots in response to the PTW treatment. Major variances within the dataset could be identified by applying PCA (Supplementary Fig. S4): Transcriptomic changes based on the two organs leaf and root correlated with the largest variation (94%). 4% of the variation within the data sets seem to be caused by plant age. Independent PCA analyses of short- and long-term effects for leaves and roots allowed an explanation of > 60% variation between treatment and control for long-term in both organs. For detailed analysis of differentially expressed genes (DEGs), we performed a DEseq2 analysis separately for leaf and root samples for each time point (Supplementary Fig. S5). Based on the distribution of log2FC and the literature [33], we decided to focus on the DEGs with an │Log2FC│ ≥ 1 and adjusted p-value < 0.05 (Supplementary Table S1) to find DEGs with the largest effects after PTW treatment.

**Fig. 1:**
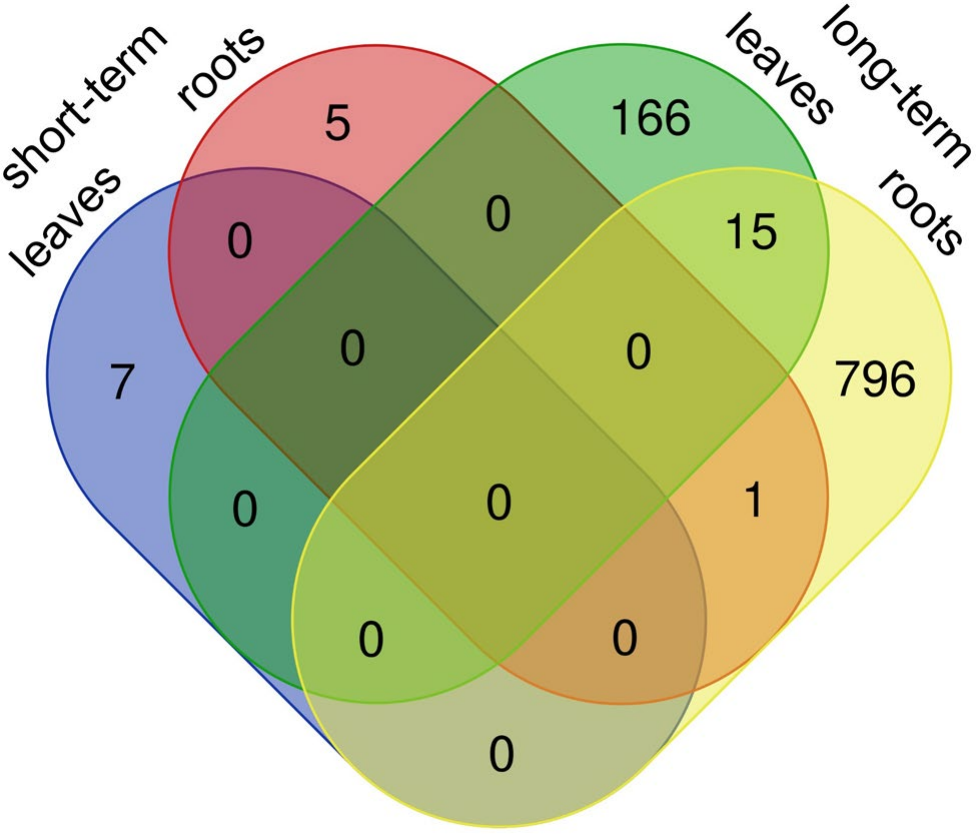
Venn diagramm representing DEGs with│Log2FC│ ≥ 1 and adjusted p-value < 0.05 between PTW-treated and control samples in barley leaves and roots one day (short-term) or four weeks (long-term) after treatment.

One day after PTW treatment (short term), only 7 DEGs were identified in the leaves and 6 DEGs in the roots (Fig. 1). In the leaves, 2 genes were upregulated and 5 were downregulated. In the roots, all 6 genes were upregulated. Four weeks after PTW treatment (long term), 181 DEGs were identified in the leaves, of which 114 were upregulated and 67 were downregulated. In the roots, 812 DEGs were detected, of which 472 were upregulated and 340 were downregulated. Among the DEGs, there was an overlap of 15 genes between leaves and roots four weeks after PTW treatment, all of which showed the same regulation in both organs (6 upregulated and 9 downregulated). Only 1 DEG (a vacuolar iron transporter-like protein) from the short-term samples was also detected in the long-term samples of root. Thus, application of PTW to the leaves resulted in largely organ-specific responses with stronger long-term transcriptional changes in the roots.

### Short-term effects of PTW treatment on DEGs in leaves and roots indicate alteration of cell wall and N-related processes

DEG analysis showed that one day after PTW treatment (short term), only 2 genes were upregulated in the leaves (Supplementary Table S1): one gene encoding an aldose epimerase (mutarotase) and one encoding an extensin. Mutarotase converts α-D-glucose to β-D-glucose and vice versa and may be involved in the conversion of sucrose to cellulose. Both genes are associated with cell wall processes [44]. Five genes were downregulated in the leaves. The downregulation of genes encoding an arabinogalactan protein and another unspecified cell wall glycoprotein again shows a short-term effect of PTW treatment on cell wall processes. Interestingly, one downregulated gene encodes a heavy metal transport protein that has a ferredoxin-like fold and could be involved in redox processes. The down-regulation of an asparaginase and an NRT gene indicates that the plants interpreted the PTW foliar spray as an N-signal, as PTW contains nitrate, nitrite and NO [26]. Asparaginase degrades asparagine to ammonia for use in amino acid biosynthesis. Thus, PTW treatment mainly affected cell wall adaptation and N metabolism in the leaves.

In the roots, 6 genes were upregulated one day after PTW treatment, 3 of which encode vacuolar iron transporter-like proteins (Supplementary Table S1). VTL2, a vacuolar iron (uptake) transporter-like protein, may be involved in redox processes together with GSTU20, which may also have dehydroascorbate reductase activity. It has also been reported that the expression of *GSTU20* was upregulated after cold plasma treatment of blueberries along with the expression of other antioxidant enzyme genes, which delayed postharvest fruit senescence [45]. The BHLH DNA-binding protein belongs to a transcription factor family. Dirigent proteins (DIR) impart stereoselectivity in the biosynthesis of lignans [46]. The barley genome displays a high variability of DIR genes involved in abiotic stress responses [47]. In cabbage, DIR are also involved in abiotic stress responses and lead to higher levels of acid-soluble lignin under waterlogging [48]. In roots, DIR assist in the assembly and lignification of the Casparian strip of the endodermis as a diffusion barrier [49]. The expression levels for 3 DIR were also increased as a long-term response in the roots (Table 4). The enhanced expression of DIR-encoding genes again shows an influence on cell wall processes.

### PTW treatment influenced different biological processes including homeostasis and signalling of phytohormones in leaves and roots in the long term

**Fig. 2:**
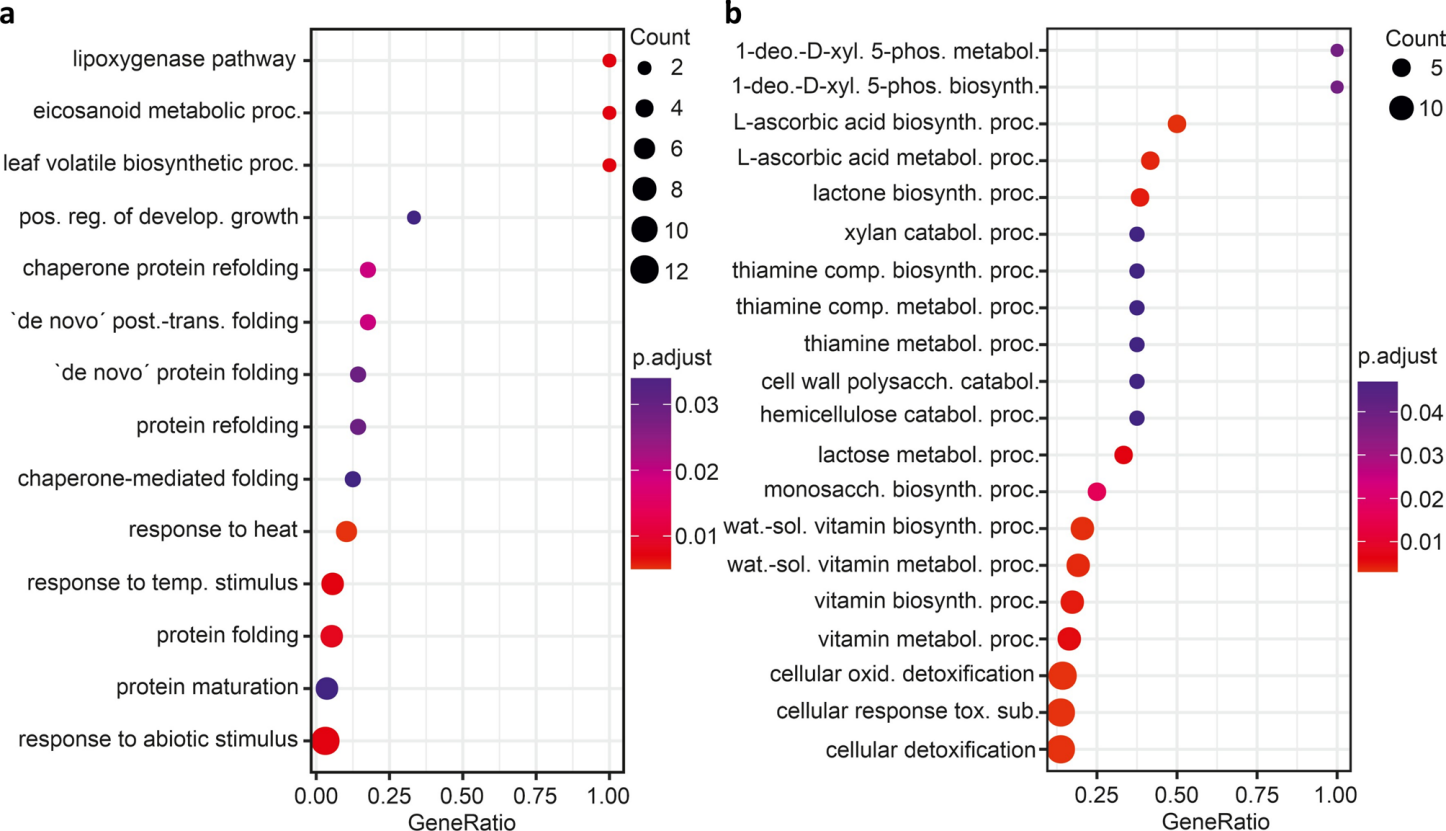
Main GO terms associated with biological processes in barley leaves (**a**) and roots (**b**) four weeks after spraying the leaves with PTW. The GO terms are based on the DEGs of PTW-treated samples compared to control samples under the premise of│Log2FC│ ≥ 1 and adjusted p-value < 0.05. Abbreviations are explained in Supplementary Table S1.

GO term analysis for biological processes showed that PTW had a different effect on leaves and roots in the long term (four weeks after PTW treatment). In leaves, the main regulated processes were the lipoxygenase pathway, positive regulation of developmental growth, protein folding and response to temperature and other abiotic stimuli (Fig. 2a). The lipoxygenase pathway (including lipoxygenase and allene oxide synthase) was upregulated. The developmental growth regulation (including nuclear transcription factor Y: up; argos-like protein: down) was regulated in both directions. Protein folding (including heat shock proteins (HSPs) and chaperones) was downregulated. Furthermore, an affected response to abiotic stimuli was observed, including the response to temperature due to the regulation of zinc-finger proteins, RNA-binding protein, plastid transcriptionally active 5 (majorly upregulated), as well as HSPs, phosphosulfolactate synthase-related protein, RNA-binding protein and dehydrin (majorly downregulated).

In roots, the L-ascorbic acid biosynthetic/metabolic process, xylan catabolic process, thiamine metabolic process, and cellular oxidant detoxification were affected (Fig. 2b). The L-ascorbic acid biosynthetic/metabolic process (L-gulonolactone oxidases) and the xylan catabolic process (including endo-1,3-β-xylanase, β-xylosidase) showed upregulation. In contrast, the response to thiamine metabolic process (including 1-deoxy-D-xylulose-5-phosphate synthase: up & down; thiamine pyrophosphokinase 1: down) and cellular oxidant detoxification (peroxidases: up; response to low sulfur protein: down) showed regulation in both directions. The vitamins ascorbate and thiamine are good candidates for communication between chloroplasts and mitochondria, acting as metabolic signals during acclimatisation [50]. The complete list of selected DEGs associated with the main biological processes in barley leaves and roots four weeks after PTW treatment is included in Supplementary Table S1.

GO term analysis for cellular compartments provided results only for roots, indicating enrichment of genes in the external encapsulating structure, cell wall and extracellular region (Supplementary Fig. S6). Together with enhanced xylan catabolism and expression of peroxidase genes, this points to a long-term effect of PTW on cell wall processes in the roots.

A number of DEGs related to phytohormone homeostasis and signalling were identified four weeks after PTW treatment. Since melatonin is considered a master regulator of phytohormones [51], DEGs related to melatonin were identified in both organs (Tables 1 and 2). In leaves, these DEGs were mainly associated with auxin, ethylene, abscisic acid (ABA) and brassinosteroids (BR) (7 genes; Table 1). In contrast, the DEGs in roots (51 genes) were mainly associated with auxin and cytokinin (Table 2). Other phytohormone-related genes in roots are shown in Supplementary Table S1.

In the leaves (Table 1), a gene encoding a 2-oxoglutarate-dependent dioxygenase (DAO1) that can inactivate indole-3-acetic acid (IAA) was upregulated, and an *HSP81.3* was downregulated. *HSP81.3* has been reported to be inducible by auxin [52]. A gene encoding an ethylene-dependent gravitropism-deficient and yellow-green-like protein (EGY3) was downregulated, while the genes encoding a GRAM domain-containing protein and a Ca^2+^-binding protein of the EF-hand family (SCS) were upregulated. EGY3 contributes to the maintenance of chloroplastic H_2_O_2_ production for retrograde signalling and is thus involved in the response to abiotic stress [53]. SCS, a Ca^2+^ sensor that interacts with SnRK2, is involved in the response to ABA [54]. A gene encoding a terpene cyclase/mutase (cycloartenol synthase CAS1) was upregulated, which is involved in BR biosynthesis [55]. An O-methyltransferase gene *(ASMT1)* was also upregulated. ASMT1 (N-acetylserotonin O-methyltransferase) is involved in melatonin biosynthesis [56]. Overall, genes encoding enzymes involved in auxin inactivation (DAO1), BR synthesis (CAS1) and ABA response were upregulated in the leaves as a long-term response to PTW treatment.

**Table 1:** DEGs associated with phytohormone homeostasis and signalling in barley leaves four weeks after PTW treatment

Gene ID	log2FC	Functional protein	Arabidopsis ortholog
Auxin			
HORVU.MOREX.r3.1HG0072100	1.03	2-oxoglutarate-dependent dioxygenase	AT1G14130 (DAO1)
HORVU.MOREX.r3.7HG0678460	−2.89	Heat shock protein 90	AT5G56010 (HSP81.3)
Ethylene			
HORVU.MOREX.r3.4HG0339340	−2.02	Ethylene-dependent gravitropism-deficient and yellow-green-like protein	AT1G17870 (EGY3)
ABA			
HORVU.MOREX.r3.6HG0597780	3.04	GRAM domain-containing protein	AT5G08350
HORVU.MOREX.r3.4HG0391600	1.24	Calcium-binding EF-hand family protein	AT4G38810 (SCS)
Brassinosteroids			
HORVU.MOREX.r3.6HG0557230	1.72	Terpene cyclase/mutase	AT2G07050 (CAS1)
Melatonin			
HORVU.MOREX.r3.7HG0747480	2.75	O-methyltransferase	AT4G35160 (ASMT1)

In the roots (Table 2), one gene for auxin biosynthesis (flavin-containing monooxygenase *YUC5*) and two genes for auxin homeostasis (IAA-amino acid hydrolase *ILL6*, IAA-amido synthetase *GH3.3*) were upregulated. GH3.3 is involved in storage, transport, compartmentalization and metabolism of IAA. ILL6 activates inactive auxin conjugates, regulates shoot and root growth and is known to be induced by NO [57]. Genes encoding an auxin efflux carrier (ABCB19) and a protein kinase (PID, positive regulator of cellular auxin efflux) were upregulated, and one gene encoding another auxin efflux carrier (PILS7) was downregulated. The influence to auxin downstream signalling was inconsistent: Genes encoding an auxin response factor (ARF) and an auxin-induced in root cultures protein 12 (a cytochrome b561/ferric reductase) were upregulated, while genes encoding a SAUR-like auxin-responsive family protein, an auxin signalling F-box protein and ARF16 were downregulated. Cytokinin metabolism tended to be downregulated, as the genes encoding a cytokinin riboside 5’-monophosphate phosphoribohydrolase (cytokinin activation pathway) and a histidine phosphotransfer protein (AHP4, positive regulator of cytokinin signalling) were downregulated, while a gene encoding a cytokinin oxidase (CKX4, cytokinin degradation pathway) was upregulated. A cytokinin-induced F-box protein-like gene *(CFB)* was also downregulated. Two genes encoding purine permeases (PUP1, PUP4), which can transport cytokinins across the plasma membrane were upregulated. With respect to ABA (Supplementary Table S1), two genes encoding ABA receptors (PYL2, PYL4) were upregulated along with genes encoding NRT1.2, a fantastic four-like protein (EAR1), a cysteine proteinase inhibitor (CYS5, phytocystatin), a glycosyltransferase (UGT75B1) and a detoxification protein (MATE45). NRT1.2 is known as an ABA importer [58]. UGT75B1 modulates ABA activity by glycosylation in response to abiotic stressors [59]. MATE45 controls the content and distribution of ABA in growing tissues [60]. Genes encoding an ABA-responsive BZIP transcription factor (AREB2), a G-box binding factor (GBF3), an abscisic stress ripening protein, a GRAM-containing/ABA-responsive protein, 2 serine/threonine protein kinases (CIPK1), another protein kinase (MAPKKK16) and a dehydration-responsive element-binding factor 1 (CBF3) were downregulated. ABA has been reported to induce the expression of *GBF3* [61] and *CBF3* [62]. CIPK1 is involved in ABA signalling [63], as is MAPKKK16 [64]. A gene encoding 1-aminocyclopropane-1-carboxylate oxidase (ACO), which is essential for ethylene biosynthesis, was upregulated along with three genes encoding ethylene-responsive transcription factors (ERFs). One *ERF* was downregulated. Regarding BR signalling genes encoding a steroid 5-alpha-reductase (DWF6, brassinolide biosynthesis), 2 brassinosteroid receptors (BRL2 and BRL3) and another receptor (NILR1) were upregulated and a protein kinase gene *(CDL1)* was downregulated. NILR1 mediates the same cascade as BRI1/BRLs (BRI1-like) and positively regulates cell expansion [65]. CDL1 positively regulates BR signalling and plant growth [66]. The genes encoding the gibberellin receptor GID1A, 2 GRAS transcription factors (TF) and 2 lectin receptor kinases (LecRK-IV.1) were upregulated. Another GRAS TF gene was downregulated. With regard to JA, the genes encoding MAPKKK14 and a jasmonate zim-domain (JAZ) protein were downregulated. MAPKKK14 is involved in plant responses to nitrogen availability [67]. In cucumber, JAZ8 downregulation promoted adventitious root formation [68]. A gene encoding ASMT1 was also upregulated in the roots. These results indicate, that auxin, ethylene, BR and gibberellin signalling tended to be stimulated in barley roots, while cytokinin signalling was inhibited and there was a differential response to ABA and JA.

**Table 2:** DEGs associated with homeostasis and signalling of auxin, cytokinin and melatonin in barley roots four weeks after PTW treatment

Gene ID	log2FC	Functional protein	Arabidopsis ortholog
Auxin			
HORVU.MOREX.r3.2HG0207720	1.28	Flavin-containing monooxygenase	AT5G43890 (YUC5)
HORVU.MOREX.r3.7HG0730040	1.33	IAA-amino acid hydrolase	AT1G44350 (ILL6)
HORVU.MOREX.r3.3HG0246970	1.04	IAA-amido synthetase GH3.3	
HORVU.MOREX.r3.2HG0173190	1.10	Auxin efflux carrier	AT3G28860 (ABCB19)
HORVU.MOREX.r3.5HG0427840	1.08	Positive regulator of cellular auxin efflux	AT2G34650 (PID)
HORVU.MOREX.r3.6HG0622400	1.70	Auxin response factor 1	
HORVU.MOREX.r3.4HG0400000	1.29	Auxin-induced in root cultures 12	AT5G48750
HORVU.MOREX.r3.7HG0731750	−1.00	Auxin response factor	AT4G30080 (ARF16)
HORVU.MOREX.r3.6HG0558630	−1.15	Auxin signaling F-box protein 1	
HORVU.MOREX.r3.5HG0494490	−1.40	SAUR-like auxin-responsive family protein	
HORVU.MOREX.r3.5HG0504260	−1.67	Auxin efflux carrier	AT5G65980 (PILS7)
Cytokinin			
HORVU.MOREX.r3.3HG0236930	1.56	Cytokinin oxidase/dehydrogenase	AT4G29740 (CKX4)
HORVU.MOREX.r3.3HG0280250	1.40	Purine permease	AT1G30840 (PUP4)
HORVU.MOREX.r3.5HG0517520	1.15	Purine permease-like protein	AT1G28230 (PUP1)
HORVU.MOREX.r3.3HG0268430	−1.18	Cytokinin riboside 5’-monophosphate phosphoribohydrolase	
HORVU.MOREX.r3.1HG0029620	−1.07	Histidine phosphotransfer protein	AT3G16360 (AHP4)
HORVU.MOREX.r3.3HG0298180	−1.10	F-box protein-like	AT2G36090 (CFB)
Melatonin			
HORVU.MOREX.r3.7HG0648350	1.33	O-methyltransferase	AT4G35160 (ASMT1)

Overall, PTW stimulated BR signalling in leaves and roots by upregulating synthesis genes *(CAS1*,* DWF6)* and genes encoding the receptors BRL2 and BRL3. BRs not only regulate vascular development in plants, particularly in controlling xylem differentiation [69], but also control cell proliferation and cell elongation [70]. *ASMT1* was stimulated by PTW in both leaves and roots. Melatonin (N-acetyl-5-methoxytryptamine) is a biostimulant and stress-reducing agent that also promotes root growth and plant development (as reviewed by Sharma et al. [71]). It can activate apoplastic peroxidases to produce hydroxyl radicals, leading to cell wall relaxation and lateral root formation [72]. Melatonin balances redox homeostasis either directly, by scavenging free radicals, or indirectly, by increasing antioxidant enzyme activities and levels of antioxidant compounds (e.g., glutathione, ascorbate, phenolic compounds and carotenoids). Here, peroxidase genes as well as L-gulonolactone oxidase genes were upregulated by PTW in the roots in the long term (Table 4; Fig. 2). The positive influence of PTW on the content of antioxidants and the activity of antioxidant enzymes has already been demonstrated [2].

### Effects of PTW treatment were stronger on roots than on leaves

**Fig. 3:**
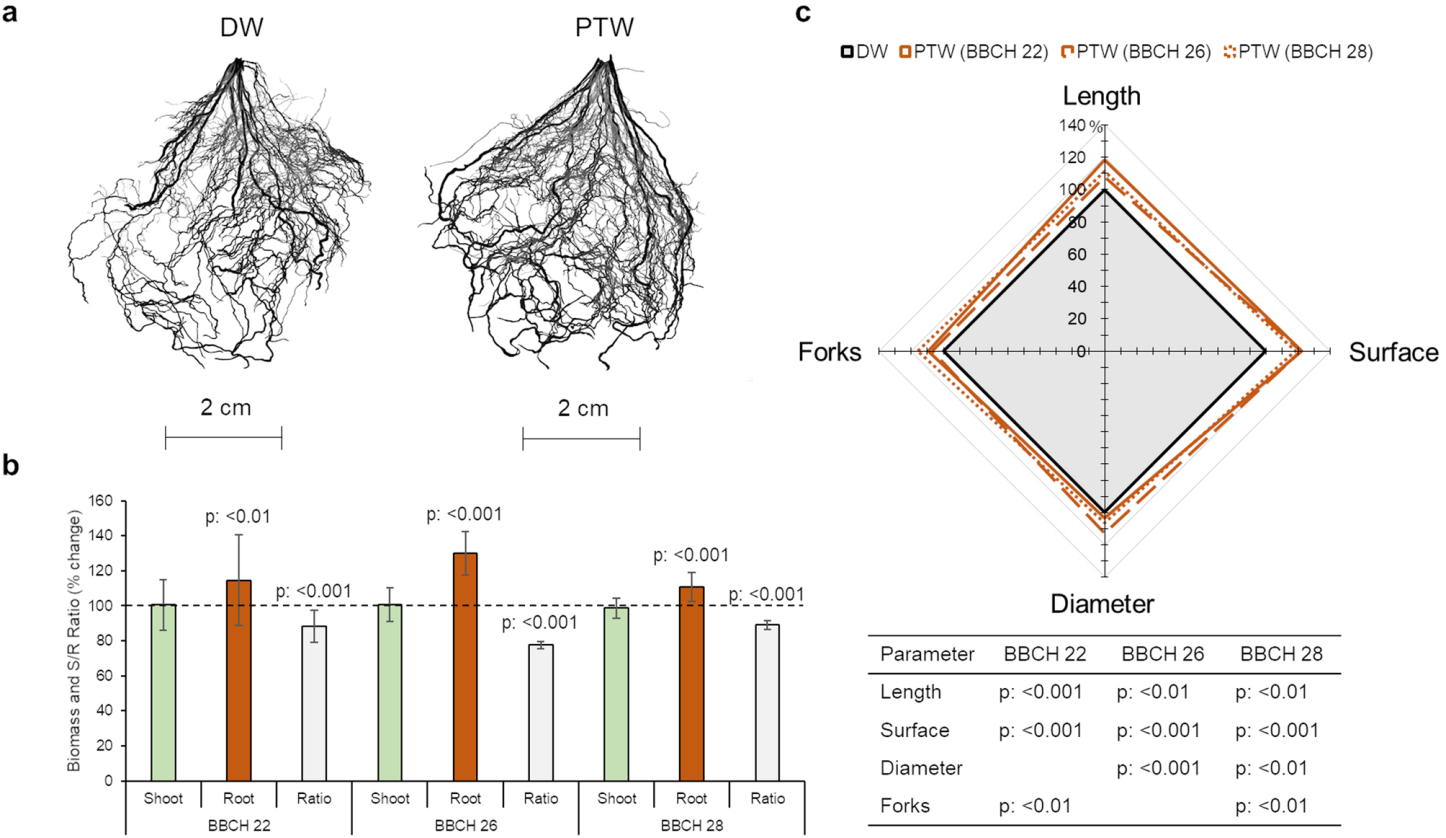
Changes in the root architecture of barley plants due to PTW treatment of leaves. Processed scans of root systems at BBCH 22 (**a**), biomass and shoot-to-root ratio (**b**). Data are shown relative to the control group (symbolized by a dashed line). Radar chart of root parameters combined with a table of statistical significance (**c**). All data shown in **b** and **c** are normalized to the control group treated with deionized water (DW; containing 7.5% (v/v) tap water, see method section). Sample size: BBCH 22 [30], BBCH 26 [10], BBCH 28 [10]. Statistical analysis was performed using student’s t-test.

To validate the influence of PTW on the roots, which had become apparent in the transcriptome analysis, we examined root system architecture in a separate experiment. Leaf treatment with PTW caused long-term changes in root architecture (Fig. 3). The roots of PTW-treated plants grew larger and became sturdier. The change in morphology was subtle but observable (Fig. 3a). PTW treatment significantly increased root biomass (Fig. 3b), while it had no effect on shoot biomass. The increase in root biomass led to a significant decrease in the shoot-to-root ratio at all growth stages. The influence of PTW on root growth was confirmed by analysing the architecture of the root system (Fig. 3c). Root length and root surface area were increased by PTW treatment at all growth stages. Significant changes in root diameter were observed at growth stages BBCH 26 and 28, while the number of root forks was increased at growth stages BBCH 22 and 28.

In supplementary experiments, the content of soluble phenolic compounds and proteins (Supplementary Fig. S7) was determined in leaves and roots, but an influence of PTW was only detected in the roots. PTW treatment increased the content of soluble phenolic compounds in roots at all growth stages studied. Phenolic compounds such as ferulic acid are essential in cell wall biosynthesis [73], with the cell wall being an important site for enzyme activities and protein incorporation [74]. The protein contents were increased by PTW only at the younger growth stages, while in the long term they approached to the values of the control group. As root cells grow, the protein content increases significantly: A threefold increase in protein content has been detected in beans [75]. The increased protein content could also be utilised as a nitrogen storage. Vegetative storage proteins serve as an N pool in the vacuole, are associated with the vascular system and their expression responds to N availability [76].

The transcriptome analysis indicated that these changes in root architecture are related to changes in cell wall processes and phenolic compound synthesis and to signal peptides. For the latter, several DEGs encoding various receptor kinases and NRTs have been identified, indicating processes related to root system architecture (RSA; Table 3).

**Table 3:** DEGs associated with signal peptides and proteins related to nutrient transport in barley roots four weeks after PTW treatment

Gene ID	log2FC	Functional protein	Arabidopsis ortholog
Signalling peptide related proteins			
HORVU.MOREX.r3.3HG0289600	2.05	Clavata3/ESR gene family member	AT2G31085 (CLE6)
HORVU.MOREX.r3.7HG0709200	2.97	Clavata3/ESR (CLE)-related protein 1	
HORVU.MOREX.r3.5HG0421040	1.38	Receptor kinase, perception of CEP	AT5G49660 (CEPR1)
HORVU.MOREX.r3.2HG0157160	1.01	Receptor kinase, perception of PSK	AT5G53890 (PSKR2)
HORVU.MOREX.r3.6HG0561600	1.19	Receptor kinase, perception of PSY1	AT1G72300 (PSY1R)
HORVU.MOREX.r3.2HG0145150	1.25	Receptor-like kinase, localizing CASPs	AT4G20140 (SGN3)
HORVU.MOREX.r3.2HG0097060	1.34	Root growth factor receptor	AT3G24240 (RGFR1)
HORVU.MOREX.r3.5HG0489130	1.01	Root growth factor receptor	AT4G26540 (RGFR3)
HORVU.MOREX.r3.2HG0186650	1.09	Serine/threonine protein kinase, mustaches-like	AT4G36180 (MUL)
Nutrient transport related proteins			
HORVU.MOREX.r3.5HG0432940	5.06	Nitrate transporter 1.2, ABA importer	
HORVU.MOREX.r3.6HG0543560	1.33	High affinity nitrate transporter 2.1	AT1G08090 (NRT2.1)
HORVU.MOREX.r3.6HG0595580	1.30	Ammonium transporter	AT4G13510 (AMT1;1)
HORVU.MOREX.r3.3HG0303830	1.11	Ammonium transporter	AT2G38290 (AMT2;1)
HORVU.MOREX.r3.1HG0041630	1.16	Peptide transporter	AT5G46050 (NPF5.2)
HORVU.MOREX.r3.1HG0075470	1.02	Peptide transporter family	AT1G22540 (NPF5.10)
HORVU.MOREX.r3.7HG0729020	−4.76	High-affinity nitrate transporter 2.2	
HORVU.MOREX.r3.4HG0334610	−1.13	Putative nitrate transporter	AT5G62720 (NITR2.1)
HORVU.MOREX.r3.5HG0529600	1.39	Phosphate transporter	AT5G43350 (PHT1;1)
HORVU.MOREX.r3.5HG0504780	−1.23	Phosphate transporter	AT5G20380 (PHT4;5)
HORVU.MOREX.r3.1HG0090860	1.30	Sulfotransferase	

Two genes encoding members of the clavata3/embryo surrounding region family (CLE6 and CLE-related) were upregulated along with genes encoding six receptor kinases (PSKR2, PSY1R, RGFR1, RGFR3, SGN3 and CEPR1) and the serine/threonine protein kinase mustaches-like (MUL). CLE6 promotes the proliferation of vascular cells [77]. CLE peptides affect cellular differentiation in a dose-dependent manner. CLE1 ~ 7 are suggested to be functionally semi-redundant, as a major role of CLE3 is to regulate auxin-mediated lateral root formation [78]. MUL is involved in the control of the early development of lateral root primordia (LRP) by regulating cell wall synthesis [79]. The receptor kinases PSKR2 and PSY1R are involved in the perception of the signal peptides phytosulfokine (PSK) and plant peptide containing sulphated tyrosine 1 (PSY1), which promote cellular proliferation and expansion [80]. PSY1 activates the transcripts of genes involved in cell wall modification [81]. PSK signalling through PSKR1 promotes root growth, primarily by increasing the size of mature cells [82]. PSK and PSY1 coordinate cell elongation and expansion in the elongation zone during root growth [83] and, together with root meristem growth factor 1 (RGF1), are required for root meristem development. The root growth factor receptors RGFR1 and RGFR3 bind RGFs, small sulphated signalling peptides that maintain cell proliferation activity in the root apical meristem [84]. Signalling through RGF and RGFR regulates the gradient formation of PLETHORA, the master regulator of root formation. The RGFR1 signalling pathway controls the distribution of RONS along the developmental zones [85]. The LRR receptor kinase SCHENGEN3 (SGN3) is the relevant receptor for Casparian strip integrity factor (CIF) responses in roots [86] and thus, necessary for Casparian strip development. SGN3 and DIR are involved in the formation of the Casparian strip [49]. SGN3 leads to localised production of RONS and lignification and strongly induces peroxidase genes [86]. CEPR1 is the receptor for the C-terminally encoded peptide (CEP), a mobile root-derived signal that is sensed in the shoot. CEP-downstream peptides (CEPDs) are synthesised in the shoot, migrate to the roots and activate the expression of nitrate transporters [87]. Genes for two ammonium transporters (AMT1;1 and AMT2;1), an NRT2.1 and an NRT1.2, two peptide transporters (NPF5.2 and NPF5.10), a sulfotransferase and a phosphate transporter (PHT1;1) were upregulated by PTW in the roots in the long term. The genes for another phosphate transporter (PHT4;5), an NRT2.2 and a putative NRT (NITR2.1) were downregulated. CEPR1 is involved in cytokinin and auxin signalling [88]. CEP signalling and its interaction with CEPR1 are known to play a role in systemic N demand signalling and root system architecture, integrating various nutrient signals (N, P, S) and coordinating whole plant growth in response to various environmental stimuli [89]. CEPDs are feedback signals between shoot and root that specifically upregulate the expression of *NRT2.1* in roots at high N levels and its post-translational activation at low N levels. CEPR1 has been shown to be a positive regulator of lateral root initiation and development [90] and is negatively regulated by CEP5. Different CEPs have different effects on root growth [91]: CEP3 arrested primary root growth, while CEP4 promoted primary and lateral root growth. RONS (H_2_O_2_ and NO) were necessary for CEP4 to promote primary root growth in cucumber. Nitrate is also involved in the control of long-term responses associated with root growth and development [87]. Nitrate and phytohormones are linked. NRT2.1, a putative nitrate sensor, is associated with ethylene signalling [92] and is involved in a pathway that integrates N and P signalling via NIGT1-clade genes [93]. NRT2.1 coordinates nutritional cues and LRP initiation, but its role is complex. Remans et al. [94] demonstrated an inhibition of LRP initiation in *atnrt2.1* mutants, while Little et al. [95] reported stimulation. The emergence of LRP is also influenced by NRT2.1 activity.

Overall, 9 genes encoding signal peptides and receptor kinases involved in root growth and cell expansion were upregulated in the roots four weeks after PTW treatment. Besides, we detected strong transcriptional regulation of several NRTs in barley roots four weeks after PTW treatment, but one NRT gene was downregulated in the leaves as early as one day after PTW treatment. Most of the contribution of signal peptides was only recognized by identifying the respective receptor kinase. However, most of the kinases in our dataset have not yet been characterized. Among all DEGs that were long-term regulated by PTW treatment, we detected 102 kinases in roots and 13 in leaves.

The other striking effect after PTW treatment could be observed in the modification of cell wall processes in the roots (Table 4). In particular, the cross-linking of phenolic compounds in plant cell walls (26 genes encoding peroxidases were upregulated) affects cell expansion and cell wall strength [96]. Genes involved in cell elongation were differentially expressed at high levels in the roots (Table 4; Supplementary Table S1). Several genes encoding cell wall related proteins were detected there, most of which were upregulated. Their function [44] includes main processes for cell wall building (one gene encoding cellulose synthase: up) and modification (14 glycosyl transferases, one β-xylanase, 2 β-xylosidases: up; one β-fructofuranosidase, one β-galactosidase, one β-glucosidase: down), which were affected by PTW treatment. Endoglucanases (2 genes encoding endo-1,4-β-glucanases, 10 genes encoding endo-1,3-β-glucanases upregulated) hydrolyse glycosidic bonds in cellulose microfibrils (CMFs) and are involved in cellulose adjustment. Pectin methylesterases (PMEs, 2 genes upregulated) can add or remove methyl groups and thus reduce or increase the cross-linking of pectins with Ca^2+^, resulting in softening or stiffening of the cell wall. PMEs also mediate the cross-linking of polysaccharides and extensins (one gene upregulated). Pectin acetylesterases (one gene downregulated) block the interactions of pectins with Ca^2+^. Pectate lyases (one gene up-, one downregulated) and polygalacturonases (PGs, 2 genes upregulated) depolymerize pectic chains, resulting in loosened walls. Further genes encoding proteins involved in pectin biosynthesis or modification were also upregulated (one polygalacturonate 4-α-galacturonosyltransferase, 2 UDP-glucuronate 4-epimerases, 5 PME inhibitors and 2 PG inhibitors). Expansins (3 genes upregulated) loosen the non-covalent bonding between CMFs and xyloglucans and, together with arabinogalactan proteins (2 genes upregulated), mediate the expansion of the cell wall. Furthermore, xyloglucan metabolism encoding genes were influenced by PTW treatment (4 xyloglucan fucosyltransferases, 5 xyloglucan galactosyltransferases, one xyloglucan endotransglucosylase/hydrolase (XTHs) upregulated; two XTHs downregulated). XTHs catalyse either the linkage of xyloglucans to cellulose (strengthening of the wall) or hydrolyse the breaking of the bonds (loosening of the wall). It has been shown that root hair density and length were positively regulated by increased expression of root developmental-related genes including XTHs in *Arabidopsis* seedlings grown in PTW [97]. These XTHs act as positive regulators of root growth through cell expansion. The authors suggested that PTW mediates root architecture and is involved in the root auxin transporter signalling system. Genes encoding proteins related to phenolic compounds, such as PAL, chalcone synthase (CHS) and 3 DIR, were upregulated in the roots. Genes encoding two chalcone isomerases (CHI) and the Ca^2+^-dependent protein kinase CPK1 were downregulated. CPK1 can phosphorylate PAL [98] and increase the activity of NADPH oxidase at the plasma membrane [99]. PAL redirects carbon flux from primary to secondary metabolism and is known to be transcriptionally regulated in response to environmental stimuli and developmental cues [100]. It has been shown in *Arabidopsis* that *PAL* transcript levels correlated with PAL activity and the accumulation of soluble phenolic compounds [101]. The total phenolic content in the roots was increased at all growth stages examined in this study (Supplementary Fig. S7).

**Table 4:** Regulation of functional proteins related to cell wall and phenolic compounds encoded by DEGs in barley roots four weeks after PTW treatment

Functional protein	up	down
Cell wall related proteins		
Arabinogalactan protein	2	
β-fructofuranosidase		1
β-galactosidase		1
β-glucosidase		1
β-xylanase	1	
β-xylosidase	2	
Cellulose synthase	1	
Cortical cell-delineating protein	7	
Endo-1,4-β-glucanase	2	
Endo-1,3-β-glucanase	10	
Expansin	3	
Extensin	1	
Glycosyl transferase	14	
Pectin acetylesterase		1
Pectin acetyltransferase	1	
Pectin methylesterase	2	
Pectin methylesterase inhibitor	5	
Pectate lyase	1	1
Peroxidase	26	
Polygalacturonase	2	
Polygalacturonase inhibiting protein	2	
Polygalacturonate 4-α-galacturonosyltransferase	1	
UDP-glucuronate 4-epimerase	2	
Xyloglucan endotransglucosylase/hydrolase	1	2
Xyloglucan fucosyltransferase	4	
Xyloglucan galactosyltransferase	5	
Phenolic compound related proteins		
Calcium-dependent protein kinase CPK1		1
Chalcone synthase	1	
Chalcone isomerase		2
Dirigent protein	3	
Phenylalanine ammonia lyase	1	

**Fig. 4:**
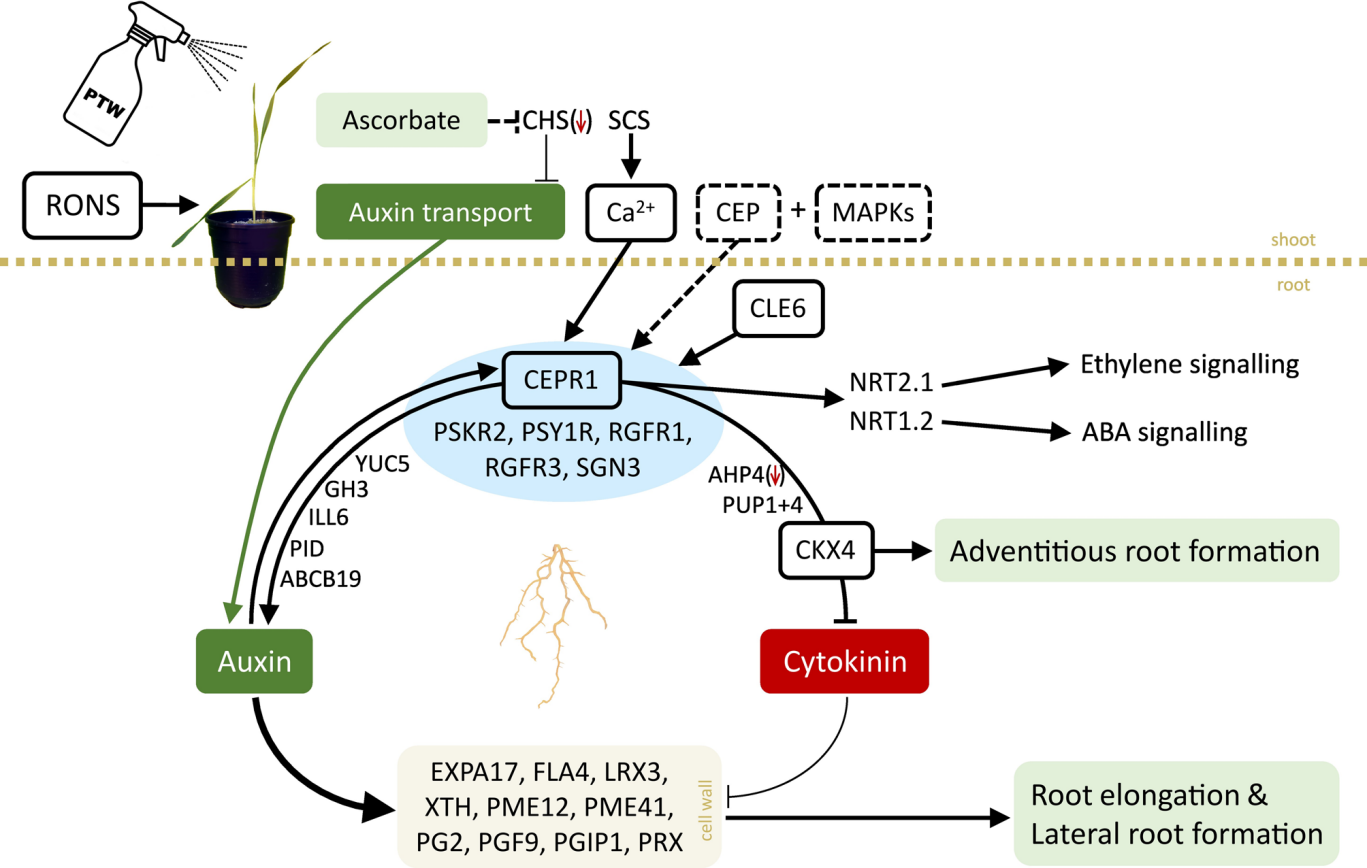
Schematic representation of the various processes regulated by PTW in barley leaves and roots as indicated by altered gene regulation, based on the model of Zhang et al. [88]. In the leaves, 3 processes could be identified converging in a concerted effect in the roots: (1) When barley leaves are sprayed with PTW (RONS), physiological processes are triggered that lead to an elevated ascorbate content in the long term. Ascorbate regulates the mRNA of CHS and probably lowers its content. CHS inhibits auxin transport to the roots, but to a lesser extent due to inactivation by ascorbate. Therefore, more auxin might be released to the root system. (2) The gene encoding SCS is upregulated, indicating calcium signalling that may be systemic. (3) MAPKs may be involved as well as CEP, which then may transmit the signal to the root. In the root, *CEPR1* is upregulated together with genes encoding other receptor kinases. CEPR1 stimulates nitrate transporters that are involved in various phytohormone signalling processes. Genes involved in auxin signalling are upregulated (transport, synthesis and deconjugation) and cytokinin signalling might be inhibited by cytokinin oxidation (upregulation of *CKX4*). In this way, genes encoding various cell wall-related enzymes and proteins are upregulated, leading to increased root growth and lateral root formation. CKX4 stimulates the formation of adventitious roots. All genes were upregulated by PTW treatment unless marked with a red arrow indicating downregulation. References are included within the text.

In the leaves cell elongation was only slightly regulated compared to the roots. Among the cell wall proteins in leaves (Table 5), genes encoding an α-galactosidase, a β-glucosidase, a cortical cell-delineating protein, two endo-1,3-β-glucanases, a GDP-mannose transporter and a peroxidase were upregulated. One pectinesterase inhibitor gene was upregulated and another was downregulated together with a glycosyltransferase gene. With regard to the synthesis of phenolic compounds, *CHS* was downregulated. CHS may be involved in the regulation of auxin transport [102]: Plants with a *tt4* mutation (CHS gene) exhibited an elevated auxin transport and showed enhanced development of secondary roots. Therefore, *CHS* expression in the leaves could be involved in the stimulated auxin response in the roots and might represent a link to improved root architecture by PTW (Figs. 3 and 4).

**Table 5:** Regulation of functional proteins related to cell wall and phenolic compounds encoded by DEGs in barley leaves four weeks after PTW treatment

Functional protein	up	down
Cell wall related proteins		
α-galactosidase	1	
β-glucosidase	1	
Cortical cell-delineating protein	1	
Endo-1,3-β-glucanase	2	
GDP-mannose transporter	1	
Glycosyltransferase		1
Peroxidase	1	
Pectinesterase inhibitor	1	1
Phenolic compound related proteins		
Chalcone synthase		1

Genes encoding cortical cell-delineating proteins were upregulated in the leaves (one) and in the roots [7] by PTW treatment. Cortical cell-delineating proteins are known to be expressed in the cortical ground meristem of maize roots [103] and accumulate near the region of fastest cellular elongation. The expression of a gene encoding a cortical cell-delineating protein increased after low dose (eustress) gamma irradiation of barley seeds, which also showed an increase in root and shoot lengths [104]. Ascorbate regulated the accumulation of mRNAs for PAL, CHS and CHI in *Arabidopsis* [105]. Here, *CHS* was downregulated in leaves, but upregulated in roots while *CHI* (2 genes) was downregulated in roots. Treatments that increase the cellular ascorbate content promote cell elongation and have an influence on the structure of the cell wall [106]. In a previous study, PTW increased the content of total and reduced ascorbate in leaves as well as the content of reduced ascorbate in roots four weeks after treatment [2]. Here, PTW treatment influenced root growth possibly by promoting cell elongation.

### Synopsis of the various effects of PTW on signalling processes in the roots

The plants treated with PTW exhibited an enlarged root surface area, which was reflected in an increase in root length and diameter (Fig. 3). Root branching also increased at later growth stages. Since we did not distinguish between different root types or developmental zones, the transcriptomic data only allow conclusions to be drawn about the entire root system, which includes both adventitious and lateral roots. It has already been proposed that the development of lateral and adventitious roots is jointly regulated [24, 107]. Recently, Omary et al. [108] discovered a conserved superlocus for root meristem initiation in the formation of adventitious and lateral roots in tomato. There is a transition state, in which a LOB-domain transcription factor named SHOOT BORNE ROOTLESS (SBRL) is transiently expressed. SBRL genes have two deeply conserved subclasses, one of which acts in the initiation of lateral roots and the other in the initiation of adventitious roots. The developmental processes of both root types are controlled by a genetic programme that determines cell fate acquisition, cell division and primordia initiation, emergence and elongation. Specific regulators that activate the common transition state create the plasticity of plant root systems.

Adventitious roots always develop from cells adjacent to vascular tissue [24]. IAA probably induces adventitious root initiation via an auxin signalling network similar to that involved in lateral root initiation as demonstrated in *Arabidopsis* [109]. Genes involved in auxin synthesis, transport and signalling were upregulated by PTW treatment in this study. Transcript accumulation and increased synthesis of the auxin transport protein ABCB19 led to elevated transport and local levels of auxin, and thus promoted adventitious root initiation in *Arabidopsis* [110]. The effect on genes involved in ethylene signalling observed in this study could possibly be due to auxin, as has been reported for rice [111]. There, auxin signalling induced ethylene biosynthesis proteins (ACO1 and ACO2) during adventitious root development by regulating the cytokinin response. This led to differential phosphorylation of cell wall proteins (cellulose synthase, UDP-glucosyltransferase). By modulating the expression of the UDP-glucosyltransferase *UGT75B1*, which was upregulated in this study and encodes the enzyme that can glucosylate and thereby inactivate PABA, the level of endogenous free PABA was manipulated and the root gravitropic response was affected [112].

The emergence of LRP depends on auxin derived from the first true leaves in *Arabidopsis* [113]. If the first leaves are also the auxin source for the adventitious and lateral roots in barley, it was these leaves that were treated with PTW in our study. The formation of a local auxin maximum leads to the initiation of LRP in the pericycle and thus, to root branching [114]. The serine/threonine protein kinase MUL, whose gene was upregulated by PTW, is involved in the control of LRP development by regulating cell wall biosynthesis and gene remodelling. *XTH*, the expansin gene *EXPA17* and *PG* were influenced by MUL [79] and upregulated by PTW in this study. The emergence of LRP is also under the control of auxin and is associated with the upregulation of genes encoding cell wall modifying enzymes such as PG and PME, as reported in this study. The same is true for the accumulation of extensins and/or arabinogalactans, which facilitate controlled cell separation in the primordium overlying cortical cells [115] and were upregulated in the long term by PTW.

Root elongation is associated with cell wall biosynthesis. Auxin stimulates cell elongation by causing cell wall loosening. The expression of genes encoding cell wall modifying enzymes is upregulated leading to the sliding of CMFs [44]. Endo-1,4-β-glucanase is necessary for root elongation in rice [107]. In this study, we found a strong upregulation of genes encoding cell wall modifying enzymes (PG, PME, XTH) and proteins by PTW treatment in the roots, which is consistent with the stimulation of auxin signalling in the long-term. CEPRs positively regulate root growth and root architecture by modulating auxin and cytokinin signalling [88] (Fig. 4).

Cytokinins negatively regulate root growth and root meristem activity. The decrease in active cytokinins as evidenced by the increased expression of *CKX4* could lead to a larger root system, probably through adventitious roots. A significant increase in the formation of adventitious roots, but not lateral roots, was observed in *CKX4* overexpression lines in *Arabidopsis* [116]. In rice, *CKX4* also positively regulated the initiation and development of adventitious roots, but had no effect on the development of lateral roots [117]. The balance between antagonistic auxin and cytokinin activities ensures proper root development by regulating cell division and differentiation in the root meristem of rice plants [118]. That was also reflected in the data of this study, where spraying the leaves with PTW stimulated the expression of genes involved in the long-term signalling of auxin (ILL6, GH3.3, YUC5, ABCB19), ethylene (ACO4) and BR (DWF6) in the roots. Genes involved in the activation and degradation of cytokinin were affected differently, indicating an attenuation of cytokinin signalling (AHP4, CKX4). Increased auxin content and decreased cytokinin content lead to a longer primary root and an increased number of lateral roots [88]. Genes encoding CEPR1 and other receptor kinases (PSKR2, PSY1R, RGFR1, RGFR3, SGN3) were upregulated in the roots four weeks after PTW treatment indicating the growth promoting effect of PTW (Fig. 4).

## Conclusion

Mild lipid peroxidation by PTW treatment as a foliar spray was accompanied by systemic transcriptional changes leading to a long-term improvement in root architecture. Root length, surface area, diameter and the number of branches increased. RNA sequencing provided evidence, that the observed changes in the root system were presumably modulated by altered concentrations and fluxes of the phytohormones auxin and cytokinin. In addition, the transcription of receptor genes for signal peptides involved in root development was affected by PTW treatment. Apparently, root growth was enhanced by transcriptomic changes in cell wall biosynthesis related proteins that mediate cell elongation.

Although our study demonstrated the systemic response of barley plants to PTW as a foliar spray, the exact details of PTW signalling are not yet clear. Future studies should investigate the influence of PTW on different root developmental zones and root types separately. Tissue-specific examinations of cuticle, endodermis and mesophyll cells after PTW application to plant leaves could also provide information on PTW signalling. PTW treatment increased the content of soluble proteins and phenolic compounds in roots, which offers an interesting perspective for further investigation. The actual influence of PTW on auxin signalling remains to be demonstrated.

## Supplementary Information

Supplementary Table S1

Supplementary Table S2

Supplementary Figures

## Data Availability

The underlying transcriptome data are available in the National Center for Biotechnology Information (NCBI) repository, https://www.ncbi.nlm.nih.gov/geo/query/acc.cgi?acc=GSE294408. Datasets supporting the conclusions of this article are included within the additional files: Supplementary_Figures.pdf (RNA gels; TBARS; PCAs; Volcano plots; GSOA for cellular compartment of the root samples; phenolic and protein contents of the root samples), Supplementary_Table_S1.xlsx (readmapping table; all DEGs short-term and long-term; GO terms; additional phytohormones regulated in the root; DEGs encoding functional proteins related to cell wall and phenolic compounds), Supplementary_Table_S2.xlsx (TBARS; biomass; RSA; phenolic and protein contents in roots).
