# Supplementary material for: Plasma-treated water applied as a foliar spray promotes root growth in barley
Source: BMC Plant Biol. 2025 Sep 22;25:1210. doi: 10.1186/s12870-025-07394-w (PMC12455771; doi:10.1186/s12870-025-07394-w)
Supplement: Supplementary file 3 — Supplementary Figures [file 12870_2025_7394_MOESM3_ESM.pdf]

Supplementary Figures to:

Plasma-treated water applied as a foliar spray promotes root growth in barley

Andrea Krüger, Stefan Schlömer, Stefan Simm, Jessica Bold, Christine Stöhr

Supplementary Figure S1: RNA gel (harvest one day after treatment)

Supplementary Figure S2: RNA gel (harvest four weeks after treatment)

Supplementary Figure S3: Lipid peroxidation

Supplementary Figure S4: PCAs

Supplementary Figure S5: Volcano plots

Supplementary Figure S6: GSOA – cellular compartment (root)

Supplementary Figure S7: Phenolic and protein contents (root)

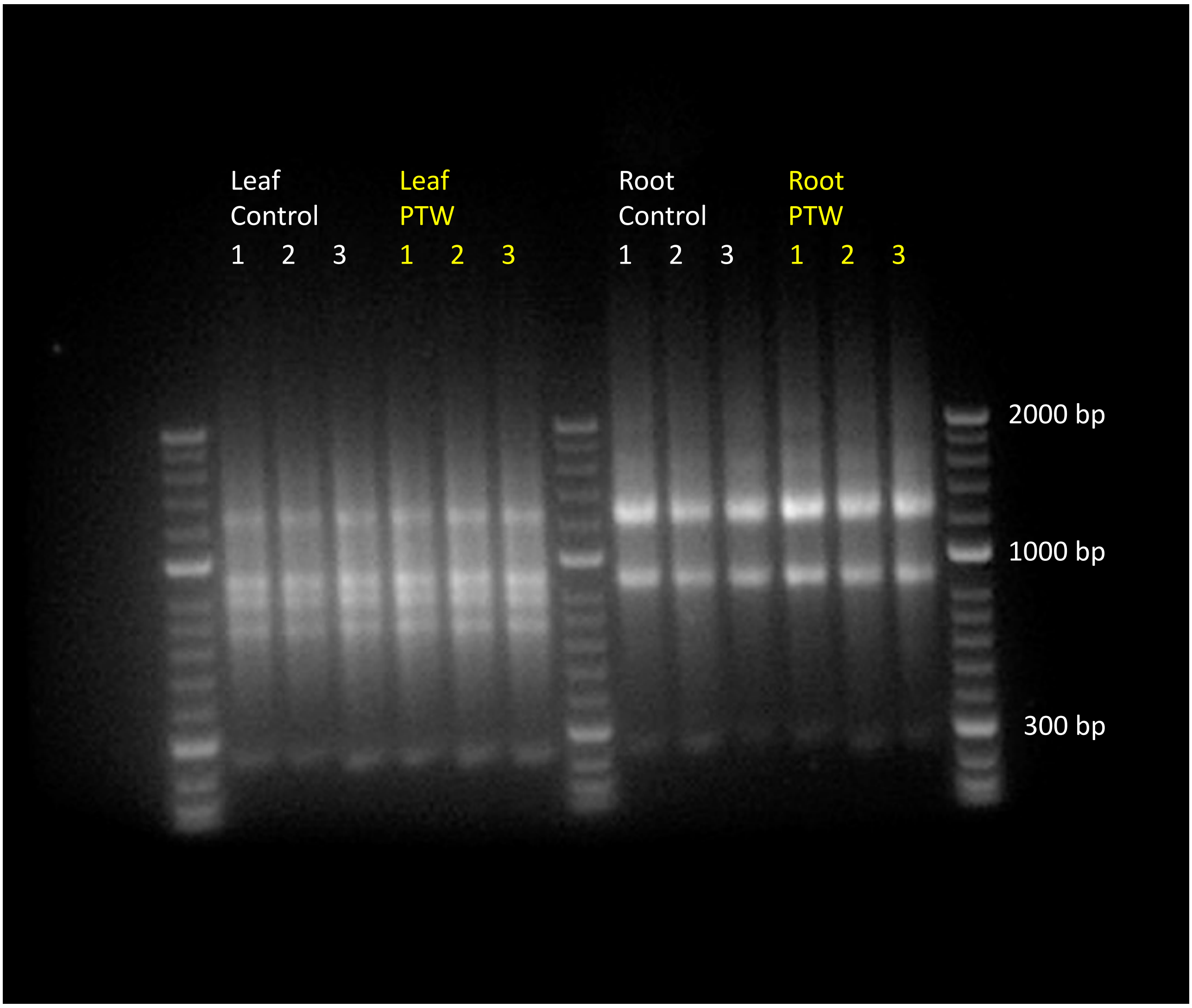

Supplementary Figure S1: RNA gel of samples harvested one day after PTW or control treatment (1 µg of total RNA per lane) and HyperLadder 50 bp (Meridian Life Science Inc., USA) on each side and in the middle lane.

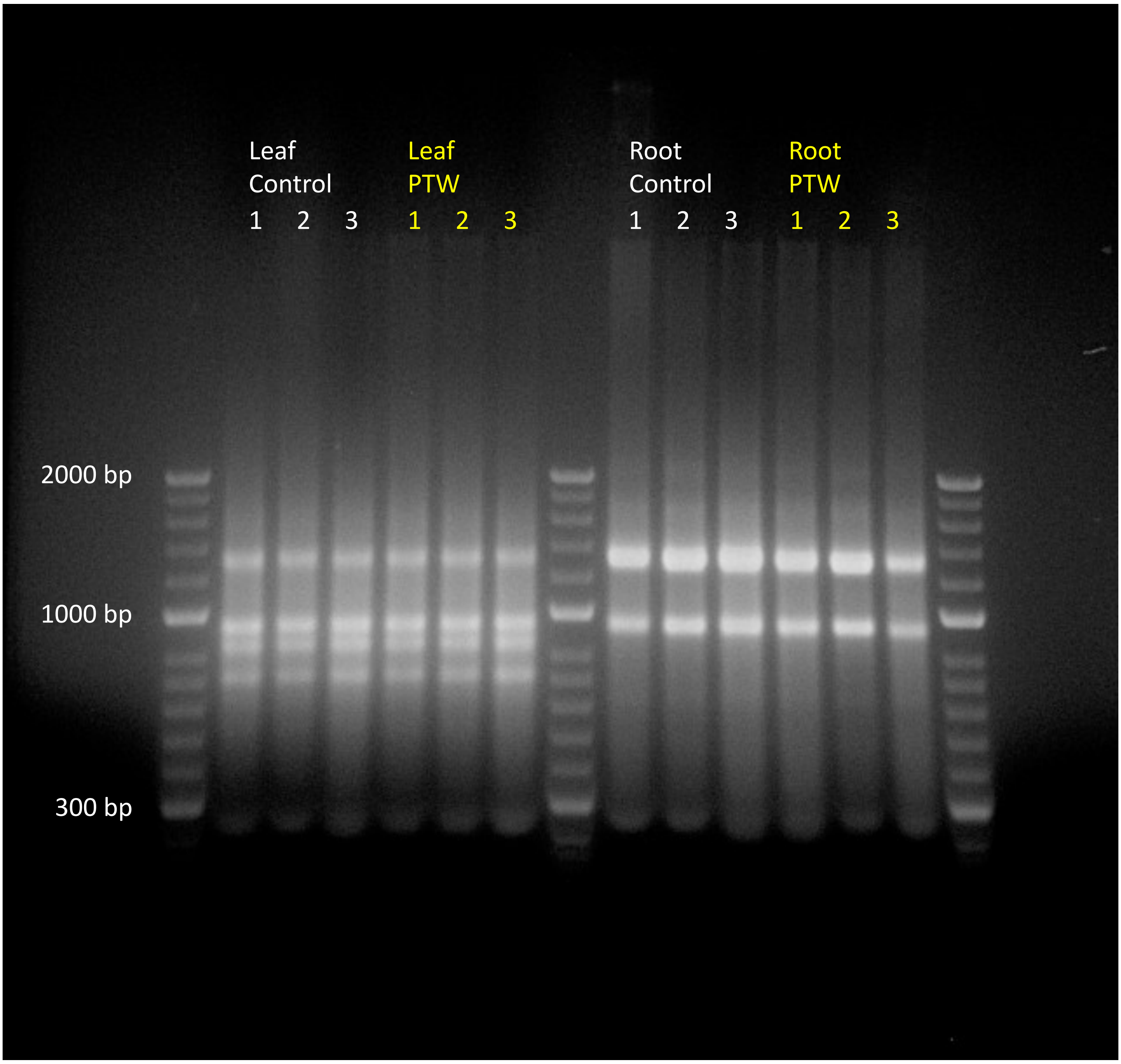

Supplementary Figure S2: RNA gel of samples harvested four weeks after PTW or control treatment (2 µg of total RNA per lane) and HyperLadder 50 bp (Meridian Life Science Inc., USA) on each side and in the middle lane.

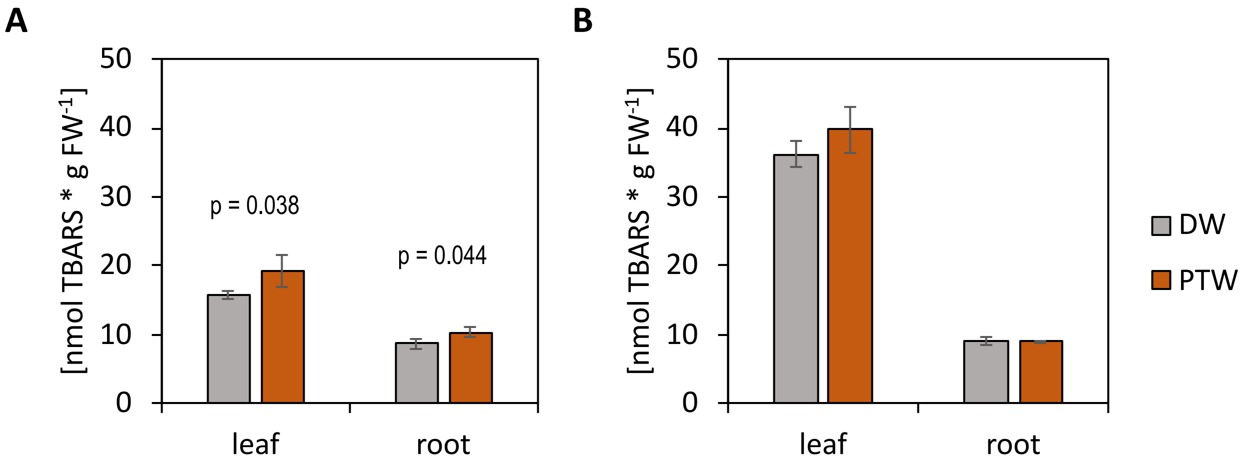

Supplementary Figure S3: Effect of PTW treatment on lipid peroxidation in barley leaves and roots. TBARS were measured one day (A) or four weeks (B) after treatment with deionized water (DW; containing 7.5% (v/v) tap water, see method section) or with plasma-treated water (PTW). Mean values (± SD) were calculated from four replicates of each treatment. Statistical analysis was performed using student's t-test.

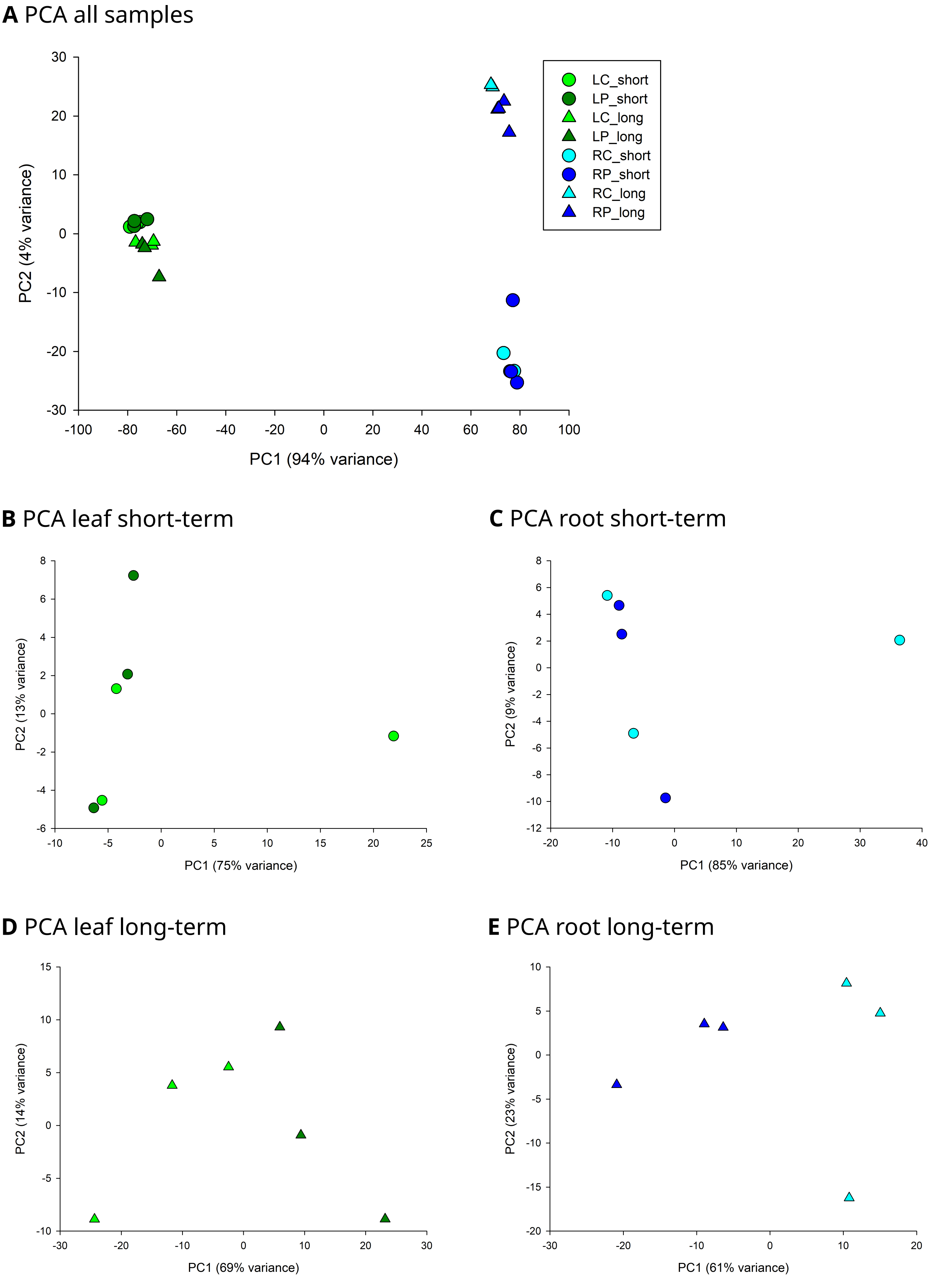

Supplementary Figure S4: Principal component analysis (PCA) of the RNA-seq data. **A** PCA of all samples. **B** PCA of the leaf samples after short-term response. **C** PCA of the root samples after short-term response. **D** PCA of the leaf samples after long-term response. **E** PCA of the root samples after long-term response. Green stands for leaf and blue for root samples with control (lighter colour) and PTW treated samples (darker colour). Circle designates short-term effects and triangle long-term effects of the respective treatment.

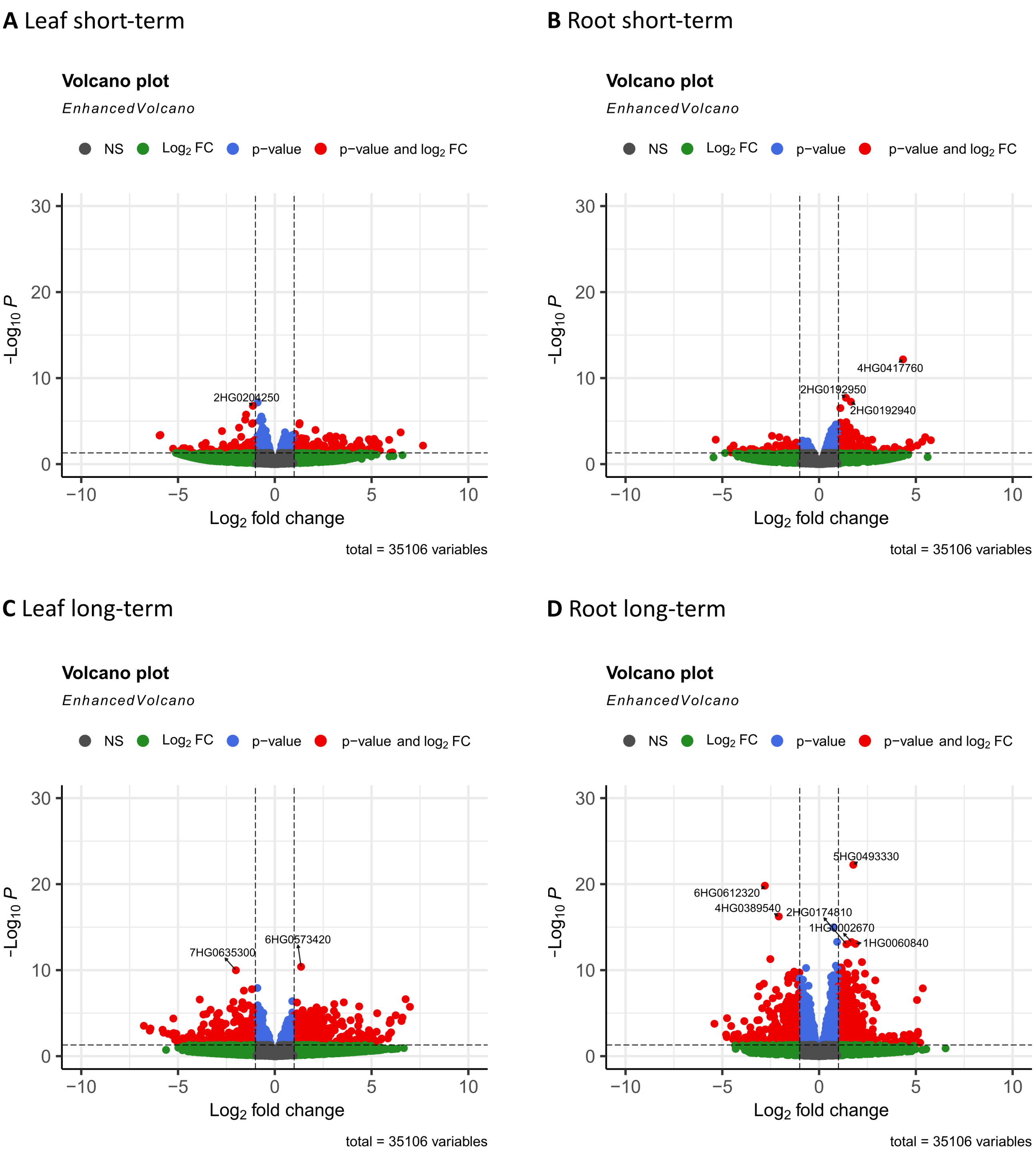

Supplementary Figure S5: Volcano plots of the differentially expressed genes between PTW-treated and control samples in **A** leaf samples and **B** root samples after short-term response, and **C** leaf samples and **D** root samples after long-term response to the treatment. The p-value threshold is 0.05 and the Log2FC is ≥|1|.

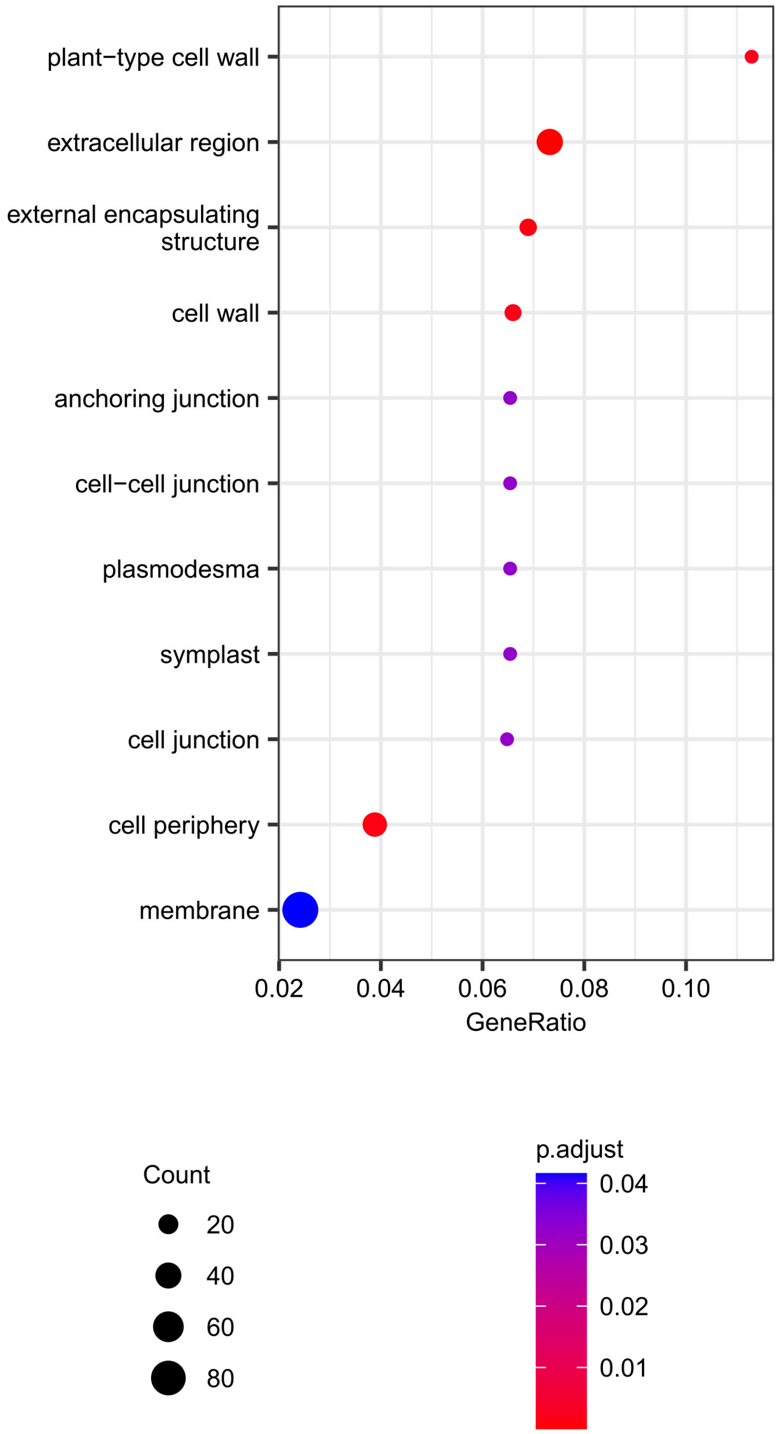

Supplementary Figure S6: Main GO terms associated with cellular compartment in barley roots upregulated four weeks after spraying the leaves with plasma-treated water (PTW). The GO terms are based on the DEGs of PTW-treated samples compared to control samples under the premise of |Log2FC| ≥ 1 and adjusted p-value < 0.05.

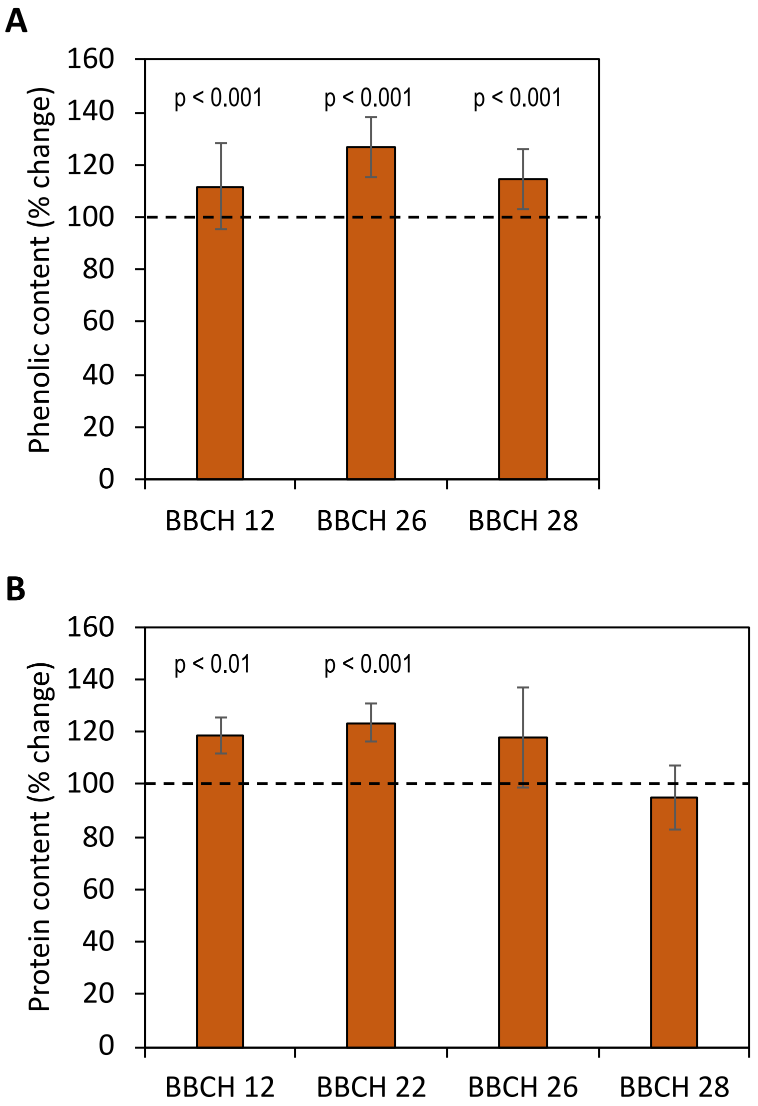

Supplementary Figure S7: Effect of PTW treatment on phenolic content (A) and protein content (B) in barley roots. All data are normalized to the control group, which is symbolized by a dashed line. Statistical analysis was performed using student's t-test.
